# Divergent complement system activation in two clinically distinct murine models of multiple sclerosis

**DOI:** 10.3389/fimmu.2022.924734

**Published:** 2022-07-26

**Authors:** Michael Linzey, Krista DiSano, Nora Welsh, Andrew Pachner, Francesca Gilli

**Affiliations:** ^1^ Department of Neurology at Dartmouth Hitchcock Medical Center, Lebanon, NH, United States; ^2^ Integrative Neuroscience at Dartmouth, Dartmouth College, Hanover, NH, United States; ^3^ Department of Veteran Affairs Medical Center, White River Junction, VT, United States

**Keywords:** multiple sclerosis, TMEV-IDD, EAE, complement system, C1q, neuroinflammation, neurodegeneration

## Abstract

Multiple sclerosis (MS) is a neurological disease featuring neuroinflammation and neurodegeneration in young adults. So far, most research has focused on the peripheral immune system, which appears to be the driver of acute relapses. Concurrently, the mechanisms underlying neurodegeneration in the progressive forms of the disease remain unclear. The complement system, a molecular component of the innate immunity, has been recently implicated in several neurological disorders, including MS. However, it is still unknown if the complement proteins detected in the central nervous system (CNS) are actively involved in perpetuating chronic inflammation and neurodegeneration. To address this knowledge gap, we compared two clinically distinct mouse models of MS: 1) proteolipid protein (PLP)-induced experimental autoimmune encephalomyelitis (rEAE) resembling a relapsing-remitting disease course, and 2) Theiler’s murine encephalomyelitis virus-induced demyelinating disease (TMEV-IDD) resembling a progressive disease. Real-time PCR was performed in the spinal cord of rEAE mice, TMEV-IDD mice and age-matched sham controls to quantify gene expression for a broad range of complement components. In both experimental models, we found significantly increased expression of complement factors, such as C1q, C3, CfB, and C3aR. We showed that the complement system, specifically the classical complement pathway, was associated with TMEV-IDD pathogenesis, as the expression of C1q, C3 and C3aR1 were all significantly correlated to a worse disease outcome (all *P*≤0.0168). In line with this finding, C1q and C3 deposition was observed in the spinal cord of TMEV-IDD mice. Furthermore, C1q deposition was detected in spinal cord regions characterized by inflammation, demyelination, and axonal damage. Conversely, activation of the classical complement cascade seemed to result in protection from rEAE (C1q: *P*=0.0307). Interestingly, the alternative pathway related to a worse disease outcome in rEAE (CFb: *P*=0.0006). Overall, these results indicate potential divergent roles for the complement system in MS. The chronic-progressive disease form is more reliant on the activation of the classic complement pathway, while protecting from acute relapses. Conversely, relapsing MS appears more likely affected by the alternative pathway. Understanding the functions of the complement system in MS is critical and can lead to better, more targeted therapies in the future.

## Introduction

Multiple sclerosis (MS) is a chronic inflammatory and neurodegenerative disease of the central nervous system (CNS) (1). MS presents with very heterogenous phenotypes resulting in highly variable individual disease pathology and clinical courses (2). There are two main disease patterns within the average MS patient population: relapsing-remitting and progressive. Relapsing-remitting MS (RRMS) is characterized by the infiltration of peripheral immune cells into the CNS associated with demyelination, neuroaxonal damage and transient neurological deficits (3).

On the other hand, progressive MS (PMS) is considered a neurodegenerative phenotype. It is clinically characterized by the accumulation of neurological disability without symptomatic recovery and pathologically described by diffuse axonal loss and CNS atrophy (4). Moreover, these pathological changes are associated with persistent compartmentalized inflammation, including the presence of activated microglia and astrocytes, i.e., resident immune and support cells of the CNS (5). However, the mechanisms of CNS damage in MS and especially PMS are not fully understood.

A shared diagnostic criterion of both RRMS and PMS is the detection of intrathecally produced immunoglobulins (Igs) in the CSF of patients. It is a prominent biological feature of MS (6) and it provides a robust marker of persistent inflammation in the intrathecal compartment (7). Notably, a growing body of evidence suggests that increased levels of intrathecally produced Igs correlate with disease progression, indicating an active, pathogenic role for Igs in MS (8). However, the actual pathogenic mechanism of these Igs is still unclear.

The complement system, also known as the complement cascade, is an essential molecular component of innate immune system. Once activated, it enhances the ability of Igs and phagocytic cells to clear pathogens, such as viruses, and damaged cells, promote inflammation, and attack the pathogen’s cell membrane (9). The complement cascade is made up of a network of proteins and cell surface receptors that recognize non-self-components and initiates one of three pathways: 1) an antigen-antibody complex that triggers the “classical pathway”, 2) foreign surfaces trigger the “alternative pathway”, and 3) carbohydrates trigger the “mannose-binding lectin (MBL) pathway” (10, 11). Each pathway is activated by a unique molecular interaction. For example, activation of the classic pathway canonically requires the complement component C1q to bind to the Fc portion of an antibody-antigen complex. All three pathways converge by forming a C3 convertase enzyme, which cleaves C3 to produce the active component C3b. The binding of a large number of C3b molecules to the pathogen/damaged cell is the main opsonizing event in complement activation (12). Continued activation of the complement cascade leads to the formation of the cytotoxic membrane attack complex (MAC) that punches holes in the cell membrane of the invading pathogen (13). Throughout this activation process, several proinflammatory molecules are released to generate an active immune response.

Historically, the complement system was thought to only defend against pathogens outside the CNS. More recently, it has been shown that complement components can also be produced within the CNS by resident glial cells, such as microglia and astrocytes (14), playing a critical role in normal synaptic pruning and neurodevelopment (15–17). The complement system has also been shown to directly control the adaptive immune responses, both B and T cells, including their recruitment in the CNS (18). Accordingly, dysregulation of the complement cascade in the CNS is associated with various neurodegenerative and neuroinflammatory diseases, including MS.

Deposition of key complement factors such as C1q, C3, and MAC have been detected in white and grey matter lesions of patients with both RRMS and PMS (16, 19–21). Complement activation can also induce microgliosis and astrogliosis in neuroinflammatory conditions and aid in the recruitment of peripheral immune cells in the CNS (22, 23). The complement cascade was also shown to inappropriately tag neuronal components, such as synapses, for removal in several animal models of neurodegenerative disorders, including models of MS (24). Therefore, it has been postulated that the complement cascade may play diverse roles in the pathogenesis of MS.

Two well-characterized animal models of MS are (1) autoimmune experimental encephalomyelitis (EAE) and (2) Theiler’s murine encephalomyelitis virus-induced demyelinating disease (TMEV-IDD). EAE is a well-established and commonly used model of autoimmune demyelination and acute neuroinflammation (25). When EAE is induced in SJL mice, using the proteolipid protein (PLP) peptide 139-151, the mice develop a severe demyelinating encephalomyelitis with a relapsing-remitting disease course (rEAE) (26). This murine model is immunologically characterized by an autoimmune response that is mediated by encephalitogenic CD4^+^ T cells. Therefore, it represents a valuable model to study the development of relapses and acute inflammation. On the other hand, TMEV-IDD is caused by direct CNS infection by the Theiler’s virus, which causes a chronic immune reaction within the CNS (27). In contrast to rEAE, B cells and antibody production play a prominent role in TMEV-IDD disease pathogenesis and progression (26, 28). Similarly, in PMS patients’ intrathecal antibody production is associated with disease progression (4, 8, 26). Moreover, TMEV-IDD shares several clinical and pathological similarities to PMS, including progressive motor impairment (29), chronic and compartmentalized neuroinflammation (30), demyelination/remyelination (27), and neuroaxonal damage (31). Finally, a viral model of MS is appropriate as recent studies have shown a strong link between viruses, such as Epstein Barr virus, and the pathogenesis of MS (32, 33).

Despite the apparent involvement of the complement system in MS pathogenesis, it is currently unknown whether its activation in the CNS differently affects the disease course. Comparing the complement activation in two clinically distinct murine models of MS could provide critical information regarding the differential involvement of the complement cascade in defining a progressive vs. relapsing-remitting disease course. Analyzing clinical outcomes, CNS gene expression of distinct complement factors and immunofluorescent imaging of spinal cords, we showed that different complement pathways are activated in the two disease models, suggesting an alternative involvement of the complement pathways in the development of an acute relapsing vs. a chronic progressive disease form.

## Methods

### Animal models of MS

The experiments consisted of the analysis of four different experimental groups of SJL/J mice: TMEV-IDD mice (n=26); sham-treated TMEV-IDD age-matched controls (n=10); PLP-immunized rEAE mice (n=20); and sham-treated rEAE age-matched controls (n=10). All SJL/J mice were purchased from The Jackson Laboratory (Bar Harbor, ME) to reduce variability. Mice were maintained on standard laboratory chow and water ad libitum, and standard clinical assessments like weight loss and clinical grading of disease were regularly performed over the follow-up.

### TMEV-IDD

TMEV-IDD was induced by injecting ten million plaque-forming units (PFU) of TMEV, strain BeAn, into the right cerebral hemisphere of 6-week-old female SJL/J mice. Mice were first anesthetized with isoflurane and inoculated by free-hand injection in a 30-μL final volume. PFUs were determined by a cytopathic effect assay (CPE).

Neurological disability was assessed weekly using the Rotamex Rotarod instrument (Columbus Instruments; Columbus, OH) and a neurological function index (NFI) as previously described by our group (29) (**Supplemental Figure 1A**).

Blood and cerebrospinal fluid (CSF) were collected from individual mice at 30- and 90-days post-infection (dpi). Blood was collected from the retro-orbital plexus of anesthetized mice; serum was then isolated and stored at −80°C. CSF was collected *via* the cisterna magna as previously described (34).

Mice were necropsied around 120 dpi, or earlier if their severe neurological disability endangered their life. Necropsy techniques, including anesthesia, intracranial injection, perfusion with phosphate-buffered saline (PBS), and the collection of blood, CSF, spinal cord and brain were performed as previously described (35).

As viral persistence in the CNS is necessary to develop TMEV-IDD (30), we only included mice chronically infected with TMEV in their CNS. Ultimately, we included 26 diseased mice out of the 34 initially infected.

### rEAE

rEAE was induced in 8-week-old SJL/J female mice by immunization with PLP_139-151_. Mice were immunized according to a standard protocol using the Hooke Kit™ PLP_139-151_/complete Freund’s adjuvant (CFA) emulsion pertussis toxin (PTx) (Hooke Labs, Lawrence, MA), which contains 200μg myelin proteolipid protein (PLP_139–151_) in 200μL CFA. The emulsion was injected subcutaneously at four sites, i.e., over the left and right shoulder and over the left and right back hip, followed by an intraperitoneal injection of 600ng of PTx in 100μL of PBS.

Mice were clinically scored blindly and daily from 7 days post-immunization (dpimm). Neurological disability was monitored using a standard EAE scoring system on a 0–5 disease severity scale. The clinical score was recorded as follows: 0, normal; 1, loss of tail tone; 2, hind limb weakness; 3, hind limb paralysis; 4, hind limb paralysis and forelimb paralysis or weakness; 5, moribund/death (36) (**Supplemental Figure 1B**).

Mice were necropsied at the peak of their first clinical episode, i.e., around 15 dpimm. CSF, serum and CNS tissues were collected as described above.

Only symptomatic rEAE mice were used for the described analyses. Ultimately, we included 20 diseased mice out of the 24 immunized.

### Control mice

Control mice were included to reflect the age differences between the rEAE and TMEV-IDD experimental mice. Thus, two distinct age- and sex-matched control groups were added. Each pairwise comparison was analyzed separately to identify unique patterns independent of age. Six-week-old TMEV-IDD sham mice received 30μL of sterile PBS *via* an intracerebral injection, while 8-week-old rEAE sham mice received four subcutaneous PBS injections. Each control group was then followed over time in parallel to the respective experimental groups.

### Time point selection for CNS tissue analysis

We selected adequate time points for tissue sampling in each model to cover the two main MS disease type, relapsing-remitting and progressive. rEAE mice were necropsied around 15 dpimm, i.e., at the peak of their first relapse, to best represent acute relapses, blood-brain barrier (BBB) leakage and immune cell infiltration. Conversely, TMEV-IDD mice were necropsied around 120 dpi, i.e., during the chronic and progressive stage of the disease, to best represent a persistent demyelinating disease characterized by neuroinflammation behind a nearly intact BBB.

### RNA extraction

Total RNA was extracted from dissected snap-frozen lumbar and sacral portions of the spinal cords with TRIzol^®^ reagent (Ambion, Austin, TX), according to the manufacturer’s protocol.

### Gene expression analyses

Complement protein C1q, complement protein 3 (C3), complement protein C3a receptor (C3aR), complement protein 5 (C5), complement protein C5a receptor (C5aR), mannose binding lectin (MBL), complement factor B (CFB), and immunoglobulin-G1 (IgG1) mRNA quantitation were performed using real-time quantitative reverse transcription polymerase chain reaction (RTqPCR). Briefly, total RNA (50 ng/μL) was reverse transcribed with the qScript™ cDNA SuperMix (QuantaBio, Gaithersburg, MD). cDNA was then used as a template for the RTqPCR analysis based on the 5′ nuclease assay with the PerfeCTa qPCR FastMix II ROX (QuantaBio). Mouse glyceraldehyde phosphate dehydrogenase (GAPDH) was used as a reference gene. TaqMan Gene Expression Assays (ThermoFisher Scientific, Waltham, MA) were used as primers and probes for C1q, C3, C3aR, C5, C5aR, MBL, CFB, and GAPDH, whereas custom primers and probes were used for the amplification of IgG1 (30). To evaluate the relative expression level of each target, we used the 2^-ΔCT^ method, in which ΔCt is calculated as Ct_target_-Ct_GAPDH_. Fold-Changes were then calculated as the normalized gene expression, i.e., 2^-ΔCT^, in the experimental sample (rEAE or TMEV-IDD) divided by the normalized gene expression in the respective age-matched control samples.

Persistent CNS infection was proven by assaying the presence of viral RNA in the spinal cord at necropsy. Absolute quantification of TMEV RNA was performed by specific 5′ nuclease RTqPCR with custom primers (37). A plasmid calibration standard curve was utilized for absolute quantification.

### Immunofluorescence

Cervical and thoracic sections of spinal cords from PBS-perfused rEAE and TMEV-IDD mice were fixed in 4% paraformaldehyde for 24 hours. Following a sucrose gradient, samples were cryopreserved in optimal cutting temperature (OCT) compound and stored at −80°C. Tissue blocks were cut in sections of 10 μm thickness. The slides were washed in 0.3% Triton 1x PBS and permeabilized for 30 minutes with 1% Triton 1x PBS. The sections were then blocked with 5% bovine serum albumin (BSA) and 10% goat serum prepared in 1x PBS. After blocking, spinal cord sections were incubated with: C1q (1:100, Abcam #Ab11861), C3 (1:100, Abcam #Ab11862), C5b-C9 (1:50, EMD Millipore #204903-1MG), IBA1 (1:500, Fujifilm Wako Pure Chemical Co. #019-19741), NeuN (1:500, Synaptic Systems #266-004), GFAP (1:500, Abcam #Ab4674), APP (1:1000, Abcam #Ab32136), and Fluoromyelin (1:300 Invitrogen #F34652). Fluoromyelin was used to detect regions of demyelination. Tissue sections were washed three times with 1x PBS and then were flooded with fluoromyelin for 20 minutes. Sections were thoroughly washed with 1x PBS. The primary antibodies were detected using the following secondary antibodies from: Goat anti-rat 488 (1:1000 Abcam #ab96887), Goat anti-Rabbit 594, (1:1000 Jackson Laboratories #103-005-155), Goat anti-chicken 594 (1:1000 Jackson Laboratories #703-585-155), and Goat anti-guinea pig 594 (1:1000 Jackson Laboratories #706-585-148). Sections were mounted with Vectashield Hardset reagent with 4′,6-diamidino-2-phenylindole (DAPI) (Vector Labs, Burlingame, CA) and examined using a Zeiss LSM 800 confocal microscope with Airyscan (Zeiss, Oberkochen, Germany). Z-series images were collected every 0.7 μm covering a tissue depth of 5–8 μm. Projected images were visualized using ImageJ software (NIH, http://rsbweb.nih.gov/ij) supplemented with the FIJI plugin set (http://fiji.sc).

### Statistics

Gene expression data were assessed using a Pearson normality tests to determine significant deviations from a normal distribution. Based on the normality results, either the parametric Student’s t test or the non-parametric Mann-Whitney U test was utilized to compare groups. The exact test used to perform analyses for each data set are denoted within the corresponding figure legend. Linear regression analysis was used to determine a relationship between gene expression and clinical outcomes. All analyses were performed using Prism version 7.00 for Windows (GraphPad, San Diego CA), and all reported *P* values were based on two-tailed statistical tests, with a significant level of 0.05.

## Results

### The complement system is activated in the CNS of both rEAE and TMEV-IDD rodents

**Figure 1:**
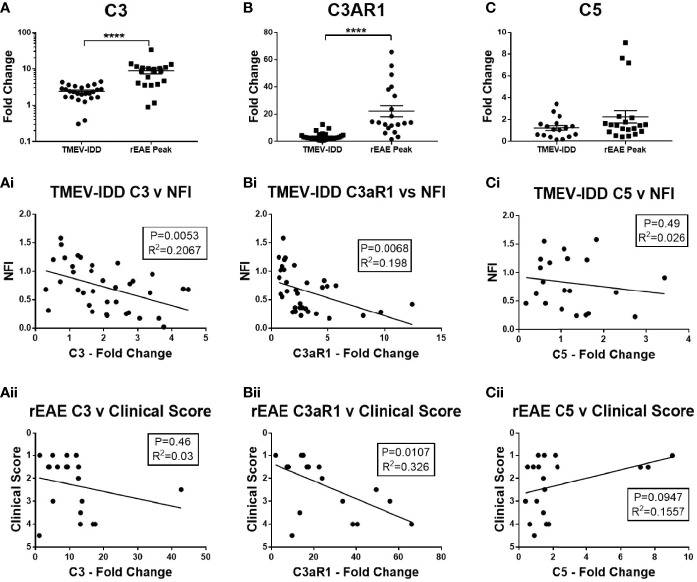
CNS gene expression of central complement components and their association with disease outcomes. Gene expression of C3, C3aR1, and C5 was determined by rt-PCR in spinal cords from TMEV-IDD mice (n = 26) at day ~120 post-infection or rEAE mice (n =20) at day ~15 post-immunization. **(A-C)** Scatter plots display C3 **(A)**, C3aR1 **(B)** and C5 **(C)** in TMEV-IDD or rEAE mice with mean ± SEM for the group. Fold changes were calculated between diseased and age-matched sham mice. Significant differences are indicated by ****(P<0.0001) as determined by Mann-Whitney U-tests. Linear regression analysis shows that high C3 gene expression **(Ai, Aii)** correlates with a worse disease outcome in TMEV-IDD, but not in rEAE, while C3aR1 **(Bi, Bii)** relates to a worse disease outcome in both rEAE and TMEV-IDD. Finally, there is no significant correlation between C5 gene expression **(Ci, Cii)** and clinical outcome neither in TMEV-IDD nor in rEAE. P value and the coefficient of determination R^2^ are indicated in each regression.

To determine whether there was local upregulation of the complement cascade in the CNS of either model, we analyzed gene expression of three key complement factors in the spinal cord, i.e., C3, C3aR, and C5. The C3 activation step represents the convergence of the lectin, classical, and alternative complement activation pathways, whereas C3aR (38) and C5 (9) play an important role in mediating neuroinflammation and neurotoxicity, respectively. Increased mRNA expression of C3 and C3aR were observed in both models, with significantly higher levels in the rEAE mice compared to the TMEV-IDD rodents (both *P*<0.0001) (**Figures 1A, B**). C3aR expression was strongly associated with a more severe disease course in both TMEV-IDD (r^2 =^ 0.198, *P*=0.0068, **Figure 1Bi**) and rEAE (r^2 =^ 0.326, *P*=0.0107, **Figure 1Bii**). Differently, increased C3 expression only correlated with a more severe disease course in TMEV-IDD (r^2 =^ 0.207, *P*=0.0053, **Figure 1Ai**). C3 was unrelated to rEAE disease outcome (**Figure 1Aii**). C5 mRNA expression was very low in both models and was not associated with disease outcomes (**Figures 1Ci, Cii**).

**Figure 2:**
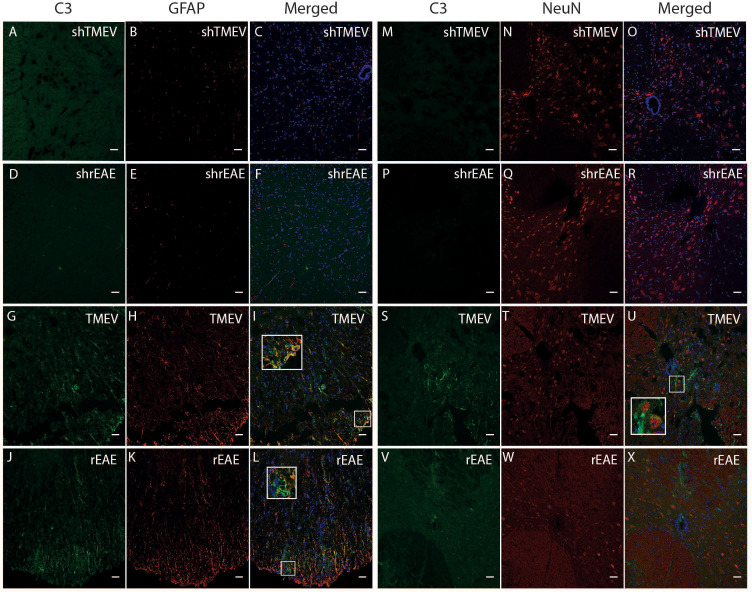
CColocalization of C3, GFAP and NeuN in the spinal cord of TMEV-IDD and rEAE mice. TMEV-IDD and rEAE mice were necropsied at ~120 dpi (chronic stage) and ~15 dpimm (acute relapse), respectively. Spinal cords were harvested for immunohistochemical analyses of the complement component C3 **(A, M, D, P, G, S, J, V)**, the astrocytic marker GFAP **(B, E, H, K)** and the neuronal marker NeuN **(N, Q, T, W)**. Age-matched sham controls **(A, D, M, P)** show no apparent C3 staining. In contrast, TMEV-IDD **(G, S)** and rEAE **(J, V)** show diffuse C3 deposits along the spinal cord. In TMEV-IDD, C3 (green) colocalizes with GFAP (red in I) and NeuN (red in **U**), confirming an association between the complement system, reactive astrocytes, and neuronal cell bodies. In rEAE, we found that C3 (green) colocalizes with GFAP (red in **L**) but not NeuN (red in **X**), confirming, in these mice, an association of the complement system with reactive astrocytes but not neuronal cell bodies. Merge of C3 and GFAP **(C, F, I, L)** and C3 and NeuN **(O, R, U, X)** is shown with DAPI (Blue) for clarity of nucleus localization. Insets in images show white boxed areas at higher magnification. Images are representative of 5-6 mice per group. Representative z stacks are shown and the scale bar = 100μM.

Given the central role of C3 in the complement cascade, we also examined spinal cord sections for deposited C3 protein. Sham mice in both age ranges showed little to no immunoreactivity throughout the spinal cord (**Figures 2A–F, M–R**). Conversely, diseased mice showed high levels of C3 depositions, with similar qualitative patterns in TMEV-IDD (**Figures 2G–I**) and rEAE mice (**Figures 2J–L**). In both models, C3 deposition appeared to occur primarily in the white matter of the spinal cord and was associated with GFAP+ cells (**Figures 2G–L**), indicating reactive astrocytes (23). C3 labeling was also associated with neuronal cell bodies in TMEV-IDD (**Figures 2S–U**), but not in rEAE mice (**Figures 2V–X**).

**Figure 3:**
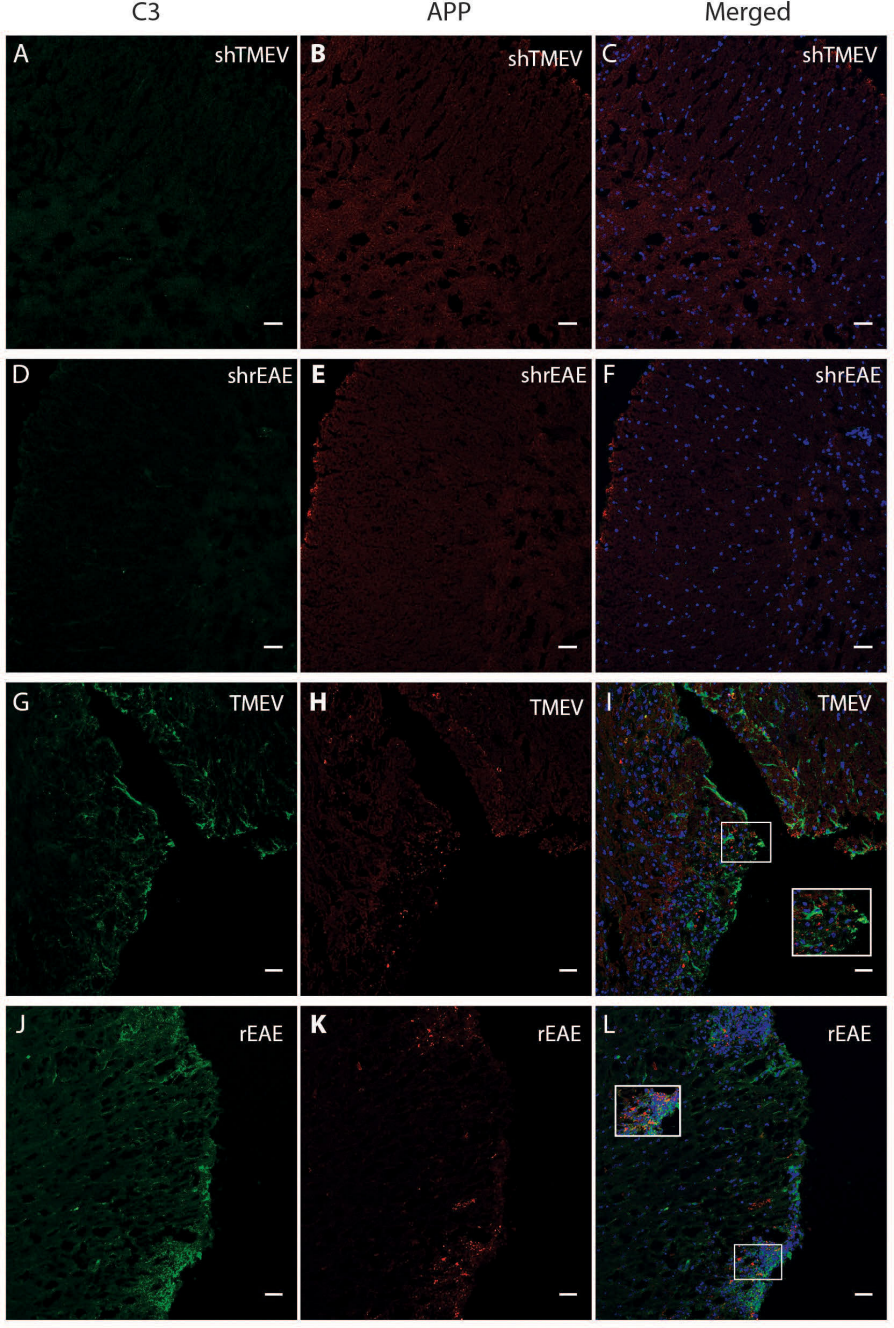
Colocalization of C3 and APP in the spinal cord of TMEV-IDD and rEAE mice. TMEV-IDD and rEAE mice were necropsied at ~120 dpi (chronic stage) and ~15 dpimm (acute relapse), respectively. Spinal cords were harvested for immunohistochemical analyses of the complement component C3 **(A, D, G, J)** and the axonal damage marker APP **(B, E, H, K)** to link complement activation and disease pathology. As expected, age-matched sham controls **(A–F)** show no obvious C3 (green) or APP (red) staining. In contrast, C3 and APP colocalize in both models **(I, L)**. Regions of interest were highlighted using inset images. Images are representative of 4-5 mice per group. Representative z stacks are shown and the scale bar = 100μM.

Based on the relationship between C3 expression and a worse disease course in TMEV-IDD, we used immunofluorescence to assess C3 deposition relative to CNS damage. We evaluated CNS damage using amyloid precursor protein (APP) accumulation, a marker of axonal injury. As expected, sham mice showed no evidence of C3 or APP immunostaining (**Figures 3A–F**). Conversely, in both models, C3 immunoreactivity was preferentially localized near regions with APP accumulation (**Figures 3G–L**).

Further immunofluorescence staining of the complement component C5b-C9 indicated little to no staining of the MAC complex, i.e., the effector of the complement terminal pathway that forms cytotoxic pores in both experimental models (**Supplementary Figure 2**). This observation is supported by the absence of increased C5 gene expression (**Figure 1C**).

### Complement activation in rEAE and TMEV-IDD occurs through different pathways

To determine the activation pathways involved in rEAE vs. TMEV-IDD, we analyzed gene expression of three early components of the complement cascade, each activating a different pathway. C1q was analyzed as the initiating protein of the classical pathway, MBL as initiating protein of the lectin pathway, and CFb as initiating protein of the alternative pathway (11).

**Figure 4:**
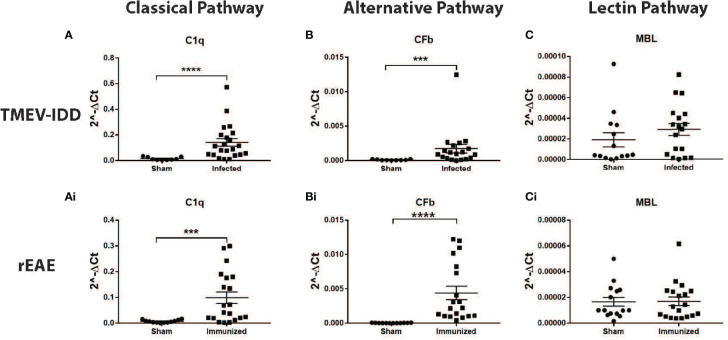
CNS gene expression of early complement components activating the three activation pathways of the complement cascade. Gene expression levels of C1q, CFb, and MBL were determined by rt-PCR in the spinal cords from TMEV-IDD mice (n = 26) at day ~120 post-infection and rEAE mice (n = 20) at day ~15 post-immunization. Age-matched sham controls were tested as reference group (n = 10 and n = 10, respectively). A significant upregulation of C1q and CFb was observed in TMEV-IDD **(A, B)** and rEAE **(Ai, Bi)** mice compared to their respective age-matched sham controls. MBL was not upregulated in either of the two models **(C, Ci)**. Scatter plots display C1q **(A, Ai)**, CFb **(B, Bi)** and MBL **(C, Ci)** in TMEV-IDD **(A–C)** or rEAE **(Ai, Bi, Ci)** mice with mean ± SEM for the group. Expression values are obtained by the ΔCt method, where GAPDH is utilized as control gene. Relative expression values (2-ΔCt) are plotted on the y-axis. Significant differences are indicated (***P<0.001 and ****P<0.0001) as determined by Mann-Whitney U-tests.

**Figure 5:**
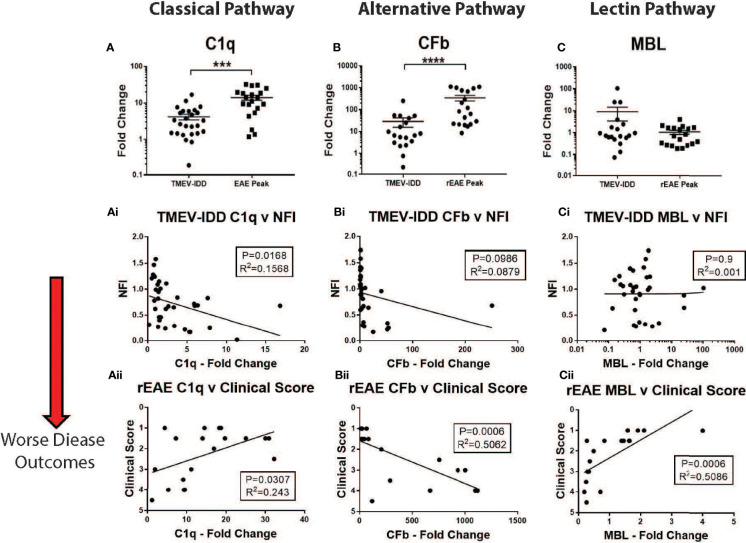
CNS gene expression of early complement components and their association with disease outcomes. Gene expression of C1q, CFb, and MBL was determined by rt-PCR in spinal cords from TMEV-IDD mice (n = 26) at day ~120 post-infection or rEAE mice (n = 20) at day ~15 post-immunization. **(A–C)** Scatter plots display C1q **(A)**, CFb **(B)** and MBL **(C)** in TMEV-IDD or rEAE mice with mean ± SEM for the group. Fold changes were calculated between diseased and age-matched sham mice. Significant differences are indicated by ****(P<0.0001) and ***(P<0.001) as determined by Mann-Whitney test. Linear regression analysis **(Ai, Aii)** shows that high C1q gene expression correlates with a worse disease outcome in TMEV-IDD (Ai), but an improved outcome in rEAE **(Aii)**. At the same time, CFb **(Bi, Bii)** relates to a worse disease outcome in rEAE, but not in TMEV-IDD. Finally, there is no significant correlation between CFb gene expression **(Ci-Cii)** and clinical outcome neither in TMEV-IDD nor in rEAE. Finally, high MBL gene expression **(Ci, Cii)** correlates with an improved clinical outcome in rEAE **(Cii)**, while no significant correlation is observed in TMEV-IDD **(Ci)**. P value and the coefficient of determination R^2^ are indicated in each regression.

We did not detect upregulation of MBL in either model (**Figures 4C, Ci**). Conversely, gene expression of both C1q and CFb was significantly increased in the spinal cords of TMEV-IDD (both *P*≤;0.0003) and rEAE mice (both *P*≤;0.0002), when compared to their respective age-matched sham controls (**Figures 4A, Ai, B, Bi**). However, both C1q and CFb were more highly expressed in the rEAE mice than in the TMEV-IDD rodents (both *P*<0.0001) (**Figures 5A, B**).

Interestingly, the expression of each activating factor was associated with different disease outcomes in each model. In TMEV-IDD, increased C1q expression was associated with a worse disease outcome (r^2 =^ 0.1568; *P*=0.0168, **Figure 5Ai**), while no significant clinical association was observed for CFb (r^2 =^ 0.0879; *P*=0.0986, **Figure 5Bi**) and MBL (r^2 =^ 0.001; *P*=0.939, **Figure 5Ci**).

Conversely, in rEAE, increased expression of C1q (r^2 =^ 0.243; *P*=0.0307, **Figure 5Aii**) correlated with an improved disease outcome, whereas CFb expression was strongly related to a worse clinical outcome (r^2 =^ 0.5062; *P*=0.0006, **Figure 5Bii**). MBL also showed a significant association with an improved outcome in rEAE (r^2 =^ 0.5086; *P*=0.0006, **Figure 5Cii**), despite not being significantly upregulated by either disease model (**Figure 5C**).

### The classical complement pathway in rEAE and TMEV-IDD

Based on the observed disease-specific increase in C1q expression in both experimental models and seemingly divergent clinical effects in the two diseases, further studies were focused on C1q and the classical pathway.

C1q is the activating factor of the classical complement pathway, usually initiated by antigen-antibody complexes with the antibody isotypes IgG and IgM (39). Previous work from our lab and others have shown that mice with chronic TMEV-IDD have, in the CNS, an abundance of IgG-secreting cells reactive primarily to Theiler’s virus and limited reactivity towards neuronal and myelin antigens (26, 37, 40). To measure intrathecally produced IgG (30), we quantified the expression levels of IgG1 mRNA in the spinal cord of TMEV-IDD and rEAE mice. The CNS expression data of Ig genes were previously shown to significantly correlate with serum anti-TMEV IgG1 IgG2b, IgG2c, and IgA antibody titers (26, 41), suggesting increased TMEV-specific Ig isotype responses explain the upregulation of Ig isotype gene expressions in the CNS. Also, we focused on IgG1 because previous work in TMEV-IDD has shown this is the predominant antibody isotype produced in the CNS, with lower intrathecal production of IgM, IgA, and IgE (26, 35).

**Figure 6:**
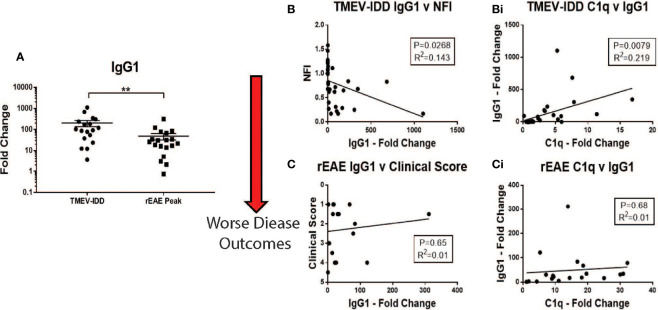
Correlations between IgG1, C1q and disease outcomes in TMEV-IDD and rEAE. Gene expression of IgG1 and C1q was determined by rt-PCR in spinal cords from TMEV-IDD mice (n = 26) at day ~120 post-infection or rEAE mice (n = 20) at day ~15 post-immunization. (A) The scatter plot displays IgG1 expression in TMEV-IDD vs. rEAE mice with mean ± SEM for the group. Fold changes were calculated between diseased and age- matched sham mice. Significant differences are indicated by **(P < 0.01) as determined by Mann-Whitney U-test. (B, Bi) Linear regression analysis shows that during TMEV-IDD, high IgG CNS expression correlates with (B) a worse disease outcome and (Bi) higher levels of C1q. In contrast, (C, Ci) during rEAE IgG1 CNS expression does not correlate with the clinical outcome or C1q levels. P value and the coefficient of determination R2 are indicated in each regression.

Correlations were calculated between IgG1, C1q and clinical outcomes for each experimental model. Mice with TMEV-IDD showed significantly higher levels of IgG1 mRNA in their spinal cords compared to the rEAE rodents (*P*=0.0061, **Figure 6A**). In TMEV-IDD, increased expression of IgG1 mRNA was significantly correlated with both a worse disease outcome (r^2 =^ 0.143, *P*=0.0268, **Figure 6B**) and increased expression of C1q (r^2 =^ 0.219; *P*=0.0079 **Figure 6Bi**). Such correlations did not extend to either the clinical scores in rEAE (r^2 =^ 0.01; *P*=0.65 **Figure 6C**) or the co-expression of IgG1 and C1q (r^2 =^ 0.01; *P*=0.68, **Figure 6Ci**).

**Figure 7:**
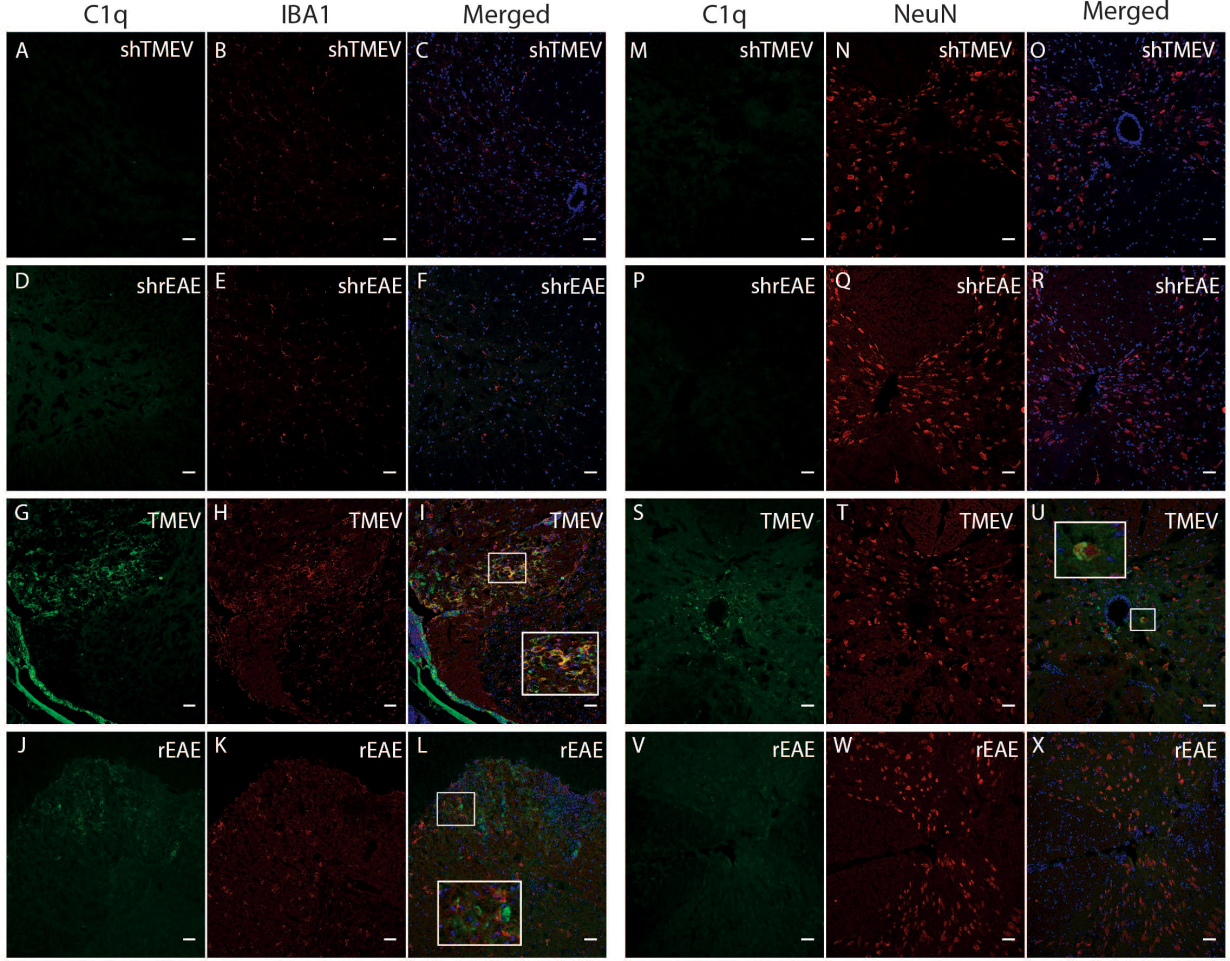
Colocalization of C1q, Iba1 and NeuN in the spinal cord of TMEV-IDD and rEAE mice. TMEV-IDD and rEAE mice were necropsied at ~120 dpi (chronic stage) and ~15 dpimm (acute relapse), respectively. Spinal cords were harvested for immunohistochemical analyses of the complement component C1q **(A, M, D, P, G, S, J, V)**, the pan-microglial marker Iba1 **(B, E, H, K, )** and the neuronal marker NeuN **(N, Q, T, W)**. Age-matched sham controls **(A–F)** show no apparent C1q or Iba1 staining. In contrast, TMEV-IDD **(H)** and rEAE **(K)** show diffuse C1q deposits along the spinal cord, mainly in the ventral lateral region. In TMEV-IDD (I, U), C1q (green) colocalizes with Iba1 (red in H) and NeuN (red in T), confirming an association between the classical complement pathway, activated microglia/macrophages, and neuronal cell bodies. Note that meninges are visualized in G, I, highlighting staining of extrinsic plasma C1q. In rEAE **(L, X)**, we found that C1q (green) does not colocalize with either Iba1 (Red in K) or NeuN (Red in W). Merge of C1q and Iba1 **(C, F, I, L)** and C1q and NeuN **(O, R, U, X)** is shown with DAPI (blue) for clarity of nucleus localization. Insets in images show white boxed areas at higher magnification. Images are representative of 5 to 6 mice per group. Representative z stacks are shown and the scale bar = 100μM.

To expand our studies beyond gene expression, we utilized immunofluorescent microscopy to localize C1q protein deposition in the spinal cord of diseased and control mice. Sham mice in each age range showed little to no C1q staining throughout the spinal cord (**Figures 7A–F, M–R**). Conversely, C1q staining was present in the spinal cord of both TMEV-IDD and rEAE mice, although qualitative differences were observed in the deposition patterns between the two experimental models. C1q staining in TMEV-IDD mice was mainly localized in the ventral lateral regions of the spinal cord (**Figure 7**). Also, in these mice C1q was shown to colocalize with Iba1+ cells (microglia/macrophages) (**Figures 7G–I**). In the central canal of the spinal cord, C1q immunoreactivity was also evident near cells expressing the neuronal nuclear protein NeuN (**Figures 7 S–U**). In contrast, in rEAE (**Figure 7**), C1q staining was not observed in areas enriched for either Iba1+ or NeuN+ cells (**Figures 7J–L, V–X**, respectively).

**Figure 8:**
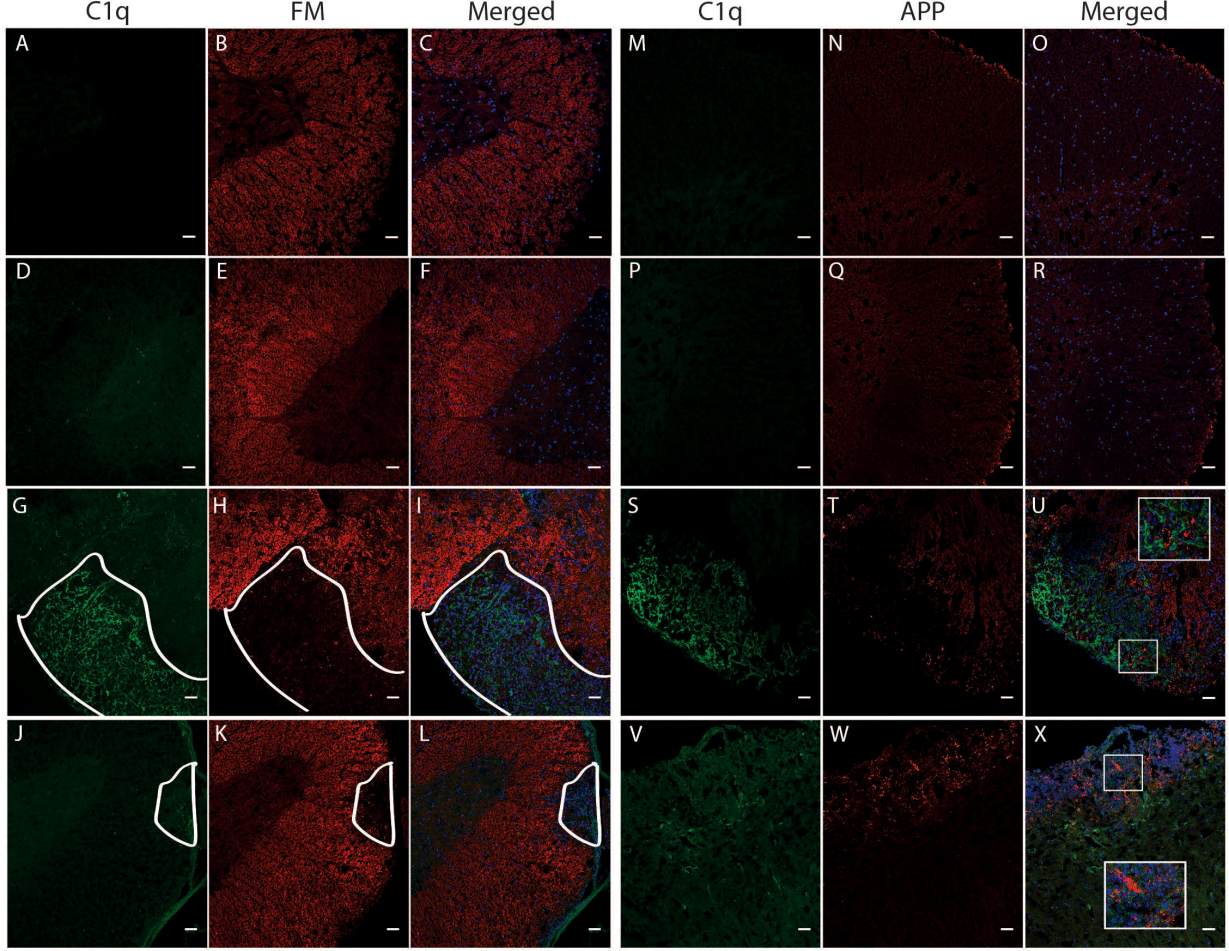
Colocalization of C1q, fluoromyelin and APP in the spinal cord of TMEV-IDD and rEAE mice. TMEV-IDD and rEAE mice were necropsied at ~120 dpi (chronic stage) and ~15 dpimm (acute relapse), respectively. Spinal cords were harvested for immunohistochemical analyses of the complement component C1q **(A, M, D, P, G, S, J, V)**, the myelin marker fluoromyelin red **(B, E, H, K)** and the axonal damage marker APP **(N, Q, T, W)**. Age-matched sham controls show no apparent C1q staining **(A, D, M, P)**, as well as no demyelination **(B, E)** or APP deposits **(N, Q)**. In TMEV-IDD, C1q staining (green in G, S) is observed in regions lacking fluoromyelin staining (red in I), i.e., demyelination and areas enriched of APP deposits (red in U), confirming an association between the activation of the classical pathway and CNS damage. In contrast, in rEAE, we found that C1q (green in J, V) does not colocalize with either demyelination (red in L) or axonal damage (red in X), suggesting that, in these mice, the activation of the classical pathway is not directly involved in CNS injury. Merge of C1q and fluoromyelin **(C, F, I, L)** and C1q and APP **(O, R, U, X)** is shown with DAPI (blue) for clarity of nucleus localization. Insets in images show white boxed areas at higher magnification. In **(G–L)**, areas of demyelination are demarcated by a solid white line. Images are representative of 4 to 5 mice per group. Representative z stacks are shown and the scale bar = 100μM.

Due to the significant correlation between C1q gene expression and a worse disease outcome in TMEV-IDD, we sought to determine if C1q deposition was associated with CNS damage, i.e., demyelination and axonal damage. As expected, both sets of sham mice showed neither demyelination (**Figures 8A–F**) nor APP deposition (**Figures 8M–R**). Conversely, demyelination and APP accumulation was evident in the spinal cords of TMEV-IDD and rEAE mice. In TMEV-IDD, C1q immunoreactivity was frequently observed in regions of demyelination (**Figures 8G–I**) and APP deposition (**Figures 8S–U**). On the contrary, in rEAE, C1q staining was only observed in areas with minimal evidence for demyelination (**Figures 8J–L**) and APP immunoreactive deposits (**Figures 8V–X**).

## Discussion

There has been a recent surge of interest in describing the effects of the complement system in neurodevelopmental and neurological disorders (9, 42). These studies indicate that the complement system likely plays a role in the pathogenesis of MS (43). However, the extent and nature of complement activation and its contribution to disease phenotype remain unclear. The above studies describe and compare complement activation in two clinically distinct murine models of MS. TMEV-IDD is a model of slowly progressing disability with many key features of a progressive demyelinating disease like PMS. At the same time, rEAE is an autoimmune inflammatory disease with an acute and relapsing disease course. Understanding the role that the complement system plays in each of these models could help design appropriate therapeutic interventions for a specific MS disease course. Although activation of the complement system has been associated with the pathogenesis of MS and its murine models, to our knowledge, few studies have been conducted to determine the differential activation of the complement system in two biologically and clinically different models of MS.

Three pathways can trigger the complement cascade, including the 1) classical pathway, which is activated by antigen-antibody immune-complexes; 2) the alternative pathway, which is activated by the spontaneous hydrolysis of the C3 component; and 3) the lectin pathway, which is activated by the recognition of certain carbohydrates on microbial surfaces (11). The three biochemical pathways ultimately converge to a common pathway where they form a crucial enzyme known as C3 convertase (44).

To assess complement activation in the two models, we first focused on the complement components C3 and C3aR. Increased mRNA levels of both factors were observed in the spinal cord of the two models, suggesting complement activity is an important contributor to each disease course. Interestingly, while C3aR expression was strongly associated with a worse disease course in TMEV-IDD and rEAE, C3 expression correlated with a worse outcome in TMEV-IDD only.

The upregulation of complement system in the CNS of TMEV-infected mice is initially due to the viral infection (45), but likely it becomes more pathogenic at a chronic stage of the disease as viral persistence over-activates the complement cascade leading to chronic inflammation and tissue damage. At this point, the complement protein expression/deposition are indeed increased on neurons and oligodendrocytes tagging them for removal by phagocytosis and driving neurodegeneration and demyelination (46). On the other hand, the activation of the complement system in rEAE is most likely linked to the autoimmune nature of the disease.

Our finding in rEAE contradicts previous reports in other EAE models showing that C3 plays a critical role in the general development and progression of EAE (24, 42, 47, 48). This may be partly due to the use of different myelin antigens in various mouse strains. Strain and immunizing antigen variations result in varying manifestations like inflammation, leukocytes infiltration, and clinical presentation, pertinent to the heterogeneous forms of MS presentation and pathology. Also, our analysis of rEAE spinal cords showed deposits of C3 protein in areas of astrogliosis, axonal damage, and demyelination. This indicates that the amount of C3 protein ultimately produced may provide sufficient activity to contribute to the development and progression of rEAE.

Surprisingly, in either model, we observed a lack of pathogenetic impact of downstream complement factors, like C5. This seems to suggest that the complement cascade plays a role in the pathogenesis of both TMEV-IDD and rEAE by stimulating phagocytes and inflammation without involving the cytotoxic action of the MAC. In accordance with this observation, we did not detect significant MAC immunoreactivity in the spinal cord of both TMEV-IDD and rEAE mice. However, it is noteworthy, that previous studies confirmed the presence of MAC deposition in the CNS lesions of MS patients (15). This discrepancy between animal models and human studies may be due to obvious limitations of preclinical studies such as heterogenicity in disease expression, a limited life span, and confounding effects of the disease.

We next analyzed the expression of three initiating complement factors, each triggering a different biochemical pathway in the complement cascade. The classical, C1q, and alternative, CFb, pathways were highly activated in both models, although activation of each pathway seemed to have a different function based on the specific disease course. Activation of the classical complement pathway was associated with a worse chronic disease course in mice with TMEV-IDD, but an improved acute disease course in mice with rEAE. One possible reason for this outcome is the different inflammatory response that characterize each model. C1q, for example, has previously been shown to label cell debris and promote phagocytosis in models of acute brain damage (49, 50). Clearance of the damaged cells and debris can aid in the resolution of inflammation and enhance endogenous repair systems, ultimately resulting in a more favorable clinical outcome. Interestingly, the pivotal role of the complement system in the handling of dying/damage cells has been mainly demonstrated for the classical pathway (via C1q) (51, 52), confirming its protective rather than damaging role in acute inflammation. However, in TMEV-IDD, a long-term progressive inflammatory and demyelinating disease, C1q is chronically activated, possibly harming the CNS by activating astrocytes and microglia and enhancing the release of pro-inflammatory cytokines (53). Co-expression of C1q on Iba1+ cells has been shown to indicate more inflammatory resident immune cells (54). Accordingly, histological analyses in TMEV-IDD revealed C1q immunoreactivity in regions of axonal damage and demyelination, as well as regions of microglia/macrophage activation. rEAE, conversely, did not have such clear patterns of C1q deposition in the CNS.

Another interesting aspect to be explored in the classical pathway activation is the prevalence of Igs in the CNS of TMEV-IDD mice. Previously we have shown increased concentrations of intrathecally produced Igs and a substantial infiltration of antibody-secreting cells (ASC) in the CNS of TMEV-IDD mice, but not rEAE mice (26, 35). This suggests a potentially pathogenic role for Igs and ASC in the chronic progressive phase of demyelinating diseases. Accordingly, in the present study, we showed increased IgG1 gene expression to be linked to increased levels of C1q as well as to a worse disease outcome in TMEV-IDD, but not in rEAE. Overall, these observations further strengthen the potential association of intrathecal synthesis of Igs, activation of the classical complement cascade, CNS injury, and TMEV-IDD disease severity. This is an important finding as it provides a possible molecular mechanism underlying CNS injury and disease progression that could be specific for chronic progressive neuroinflammatory conditions. Clearly, it is unlikely that the classic complement pathway is solely responsible for the disease course witnessed in TMEV-IDD. However, our current study was not designed to assess the role of the complement cascade or its different components in CNS inflammation/damage. Further functional studies are required to elucidate the mechanism(s) of complement-mediated injury and determine if C1q inhibition will be therapeutically beneficial in TMEV-IDD and ultimately in PMS.

Our data showed that activation of the alternative pathway related to the severity of the acute disease seen in rEAE, but not in TMEV-IDD. This result agrees with previous research findings showing that the complement system contributes to the development of EAE, and that the alternative pathway rather than the classical pathway is vital in the development of the disease (24). Such evidence is supported by studies in C3 deficient mice showing an attenuated EAE phenotype (55), and C4 deficient rodents, i.e., mice lacking the classical pathway, showing EAE onset identical to wild-type animals (56).

Taken together, our results reveal an association between demyelinating diseases and activation of the complement cascade. However, different pathways of activation seem to drive distinct disease courses. Activation of the classical complement pathway by antigen–antibody immune complexes appear to contribute significantly to disease severity in a chronic progressive model like TMEV-IDD. Conversely, activation of the alternative pathway seems to contribute to an acute, relapsing disease course like rEAE. This may indicate that the type of event, acute or chronic, may utilize different complement pathways as a mechanism of disease progression.

## Conclusion

In conclusion, the complement system plays a critical role regarding the disease course of two clinically distinct models of MS. Activation of the classical complement pathway was strongly associated with disease progression in TMEV-IDD, a model of chronic progressive MS. On the other hand, activation of the alternative pathway was more relevant in rEAE, a model of acute, relapsing MS. These results provide a proof-of-concept for exploring C1q-targeted treatment options in PMS, a disease state where there are very limited and minimally effective therapies. Targeting of C1q specifically inhibits the classical complement pathway and could provide a unique target for further development.

## Data availability statement

The original contributions presented in the study are included in the article/**supplementary material**. Further inquiries can be directed to the corresponding author.

## Ethics statement

The animal study was reviewed and approved by IACUC Committee at the Trustees of Dartmouth College.

## Author contributions

Study design and conceptualization: ML, FG, and AP. The experimental protocols were carried out by ML, KD, and NW. The data analysis was performed by ML and FG. Original draft preparation was performed by ML and FG. Data interpretation and draft revisions were performed by all listed authors. All contributing authors have approved the submitted version. All authors contributed to the article and approved the submitted version.

## Funding

This research was funded by research grants from the Hitchcock Foundation, the Diamond endowment, and the Bornstein Research Fund.

## Acknowledgments

The authors thank the staff of the Center for Comparative Medicine and Research (CCMR) at Dartmouth College for their expert care of the mice used for this study. The authors also acknowledge the NCCC light microscopy shared resource (IPIM) supported in part by NCI Cancer Center Support Grant 5P30 CA023108-37 and also NIH S10 SIG award 1S10OD021616-01 funding the LSM800 confocal.

## Conflict of interest

FG and ARP have received research support from Biogen Idec, Sanofi Genzyme, and Serono.

The remaining authors declare that the research was conducted in the absence of any commercial or financial relationships that could be construed as a potential conflict of interest.

## Publisher’s note

All claims expressed in this article are solely those of the authors and do not necessarily represent those of their affiliated organizations, or those of the publisher, the editors and the reviewers. Any product that may be evaluated in this article, or claim that may be made by its manufacturer, is not guaranteed or endorsed by the publisher.

## Supplementary material

Supplementary Figure 1 Clinical disease progression in TMEV-IDD and rEAE mice included in this study. Disease progression of TMEV-IDD **(A)** and rEAE **(B)** over their respective monitor periods post-induction. Each plot indicates the time points chosen for analysis of CNS tissue: TMEV-IDD mice were necropsied at around 120 dpi to capture the chronic progressive phase of the disease. rEAE mice were necropsied around 15 dpimm to capture the acute phase of the first relapse. **(A)** The Rotarod Test was performed on 26 TMEV-infected mice and 10 age-matched sham controls over an average of 120 dpi. Eight were excluded from the experiment because they tested TMEV-negative at the end of their follow-up. Rotarod data were expressed as a neurological function index (NFI) (29). **(B)** Clinical symptoms score (CSS) from day 1 to day 15 dpimm of 20 rEAE mice. Four mice were withdrawn from the experiment because not symptomatic at the end of the follow up. The circles and confidence bars represent the mean and the SEM of the **(A)** NFI or **(B)** CSS of the entire group of mice for each day p.i. Blue circles represent diseased mice and orange squares represent sham-treated controls.

Supplementary Figure 2 Colocalization of C5b-9 and NeuN in the spinal cord of TMEV-IDD and rEAE mice. TMEV-IDD and rEAE mice were necropsied at ~120 dpi (chronic stage) and ~15 dpimm (acute relapse), respectively. Spinal cords were harvested for immunohistochemical analyses of the late complement component C5b-9 and the neuronal marker NeuN. In both **(A)** rEAE and **(B)** TMEV-IDD mice, we found that C5b-9 (green) does not colocalize with NeuN (red), suggesting that, in these mice, there is no triggering of the cytotoxic component of the complement system. Images are representative of 3 to 4 mice per group. Representative z stacks are shown and the scale bar=100µM.

## References

[1] Disanto G Berlanga AJ Handel AE Para AE Burrell AM Fries A. Heterogeneity in multiple sclerosis: scratching the surface of a complex disease. Autoimmune Dis (2010) 2011:932351. doi: 10.4061/2011/932351 21197462 PMC3005811

[2] Katz Sand I. Classification, diagnosis, and differential diagnosis of multiple sclerosis. Curr Opin Neurol (2015) 28(3):193–205. doi: 10.1097/WCO.0000000000000206 25887774

[3] Madsen C. The innovative development in interferon beta treatments of relapsing-remitting multiple sclerosis. Brain Behav (2017) 7(6):e00696. doi: 10.1002/brb3.696 28638705 PMC5474703

[4] Correale J Gaitan MI Ysrraelit MC Fiol MP. Progressive multiple sclerosis: from pathogenic mechanisms to treatment. Brain. (2017) 140(3):527–46. doi: 10.1093/brain/aww258 27794524

[5] Lassmann H van Horssen J Mahad D. Progressive multiple sclerosis: pathology and pathogenesis. Nat Rev Neurol (2012) 8(11):647–56. doi: 10.1038/nrneurol.2012.168 23007702

[6] Beseler C Vollmer T Graner M Yu X. The complex relationship between oligoclonal bands, lymphocytes in the cerebrospinal fluid, and immunoglobulin G antibodies in multiple sclerosis: Indication of serum contribution. PloS One (2017) 12(10):e0186842. doi: 10.1371/journal.pone.0186842 29059249 PMC5653326

[7] Farina G Magliozzi R Pitteri M Reynolds R Rossi S Gajofatto A. Increased cortical lesion load and intrathecal inflammation is associated with oligoclonal bands in multiple sclerosis patients: a combined CSF and MRI study. J neuroinflamma (2017) 14(1):40. doi: 10.1186/s12974-017-0812-y PMC5319028 28222766

[8] Gasperi C Salmen A Antony G Bayas A Heesen C Kumpfel T. Association of intrathecal immunoglobulin G synthesis with disability worsening in multiple sclerosis. JAMA Neurol (2019) 76(7):841–9. doi: 10.1001/jamaneurol.2019.0905 PMC6583696 31034002

[9] Dalakas MC Alexopoulos H Spaeth PJ. Complement in neurological disorders and emerging complement-targeted therapeutics. Nat Rev Neurol (2020) 16(11):601–17. doi: 10.1038/s41582-020-0400-0 PMC7528717 33005040

[10] Alexander JJ Anderson AJ Barnum SR Stevens B Tenner AJ. The complement cascade: Yin-yang in neuroinflammation–neuro-protection and -degeneration. J Neurochem (2008) 107(5):1169–87. doi: 10.1111/j.1471-4159.2008.05668.x PMC4038542 18786171

[11] Merle NS Church SE Fremeaux-Bacchi V Roumenina LT. Complement system part I - molecular mechanisms of activation and regulation. Front Immunol (2015) 6:262. doi: 10.3389/fimmu.2015.00262 26082779 PMC4451739

[12] Merle NS Noe R Halbwachs-Mecarelli L Fremeaux-Bacchi V Roumenina LT. Complement system part II: Role in immunity. Front Immunol (2015) 6:257. doi: 10.3389/fimmu.2015.00257 26074922 PMC4443744

[13] Morgan BP Chamberlain-Banoub J Neal JW Song W Mizuno M Harris CL. The membrane attack pathway of complement drives pathology in passively induced experimental autoimmune myasthenia gravis in mice. Clin Exp Immunol (2006) 146(2):294–302. doi: 10.1111/j.1365-2249.2006.03205.x 17034582 PMC1942050

[14] Veerhuis R Nielsen HM Tenner AJ. Complement in the brain. Mol Immunol (2011) 48(14):1592–603. doi: 10.1016/j.molimm.2011.04.003 PMC3142281 21546088

[15] Ingram G Loveless S Howell OW Hakobyan S Dancey B Harris CL. Complement activation in multiple sclerosis plaques: an immunohistochemical analysis. Acta neuropathol Commun (2014) 2:53. doi: 10.1186/2051-5960-2-53 24887075 PMC4048455

[16] Watkins LM Neal JW Loveless S Michailidou I Ramaglia V Rees MI. Complement is activated in progressive multiple sclerosis cortical grey matter lesions. J neuroinflamma (2016) 13(1):161. doi: 10.1186/s12974-016-0611-x PMC4918026 27333900

[17] Stevens B Allen NJ Vazquez LE Howell GR Christopherson KS Nouri N. The classical complement cascade mediates CNS synapse elimination. Cell. (2007) 131(6):1164–78. doi: 10.1016/j.cell.2007.10.036 18083105

[18] Killick J Morisse G Sieger D Astier AL. Complement as a regulator of adaptive immunity. Semin Immunopathol (2018) 40(1):37–48. doi: 10.1007/s00281-017-0644-y 28842749 PMC5794818

[19] Vercellino M Marasciulo S Grifoni S Vallino-Costassa E Bosa C Pasanisi MB. Acute and chronic synaptic pathology in multiple sclerosis gray matter. Mult Scler (2022) 28(3):369–82. doi: 10.1177/13524585211022174. 34124960

[20] Storch MK Piddlesden S Haltia M Iivanainen M Morgan P Lassmann H. Multiple sclerosis: *in situ* evidence for antibody- and complement-mediated demyelination. Ann Neurol (1998) 43(4):465–71. doi: 10.1002/ana.410430409 9546327

[21] Lassmann H Brück W Lucchinetti C. Heterogeneity of multiple sclerosis pathogenesis: implications for diagnosis and therapy. Trends Mol Med (2001) 7(3):115–21. doi: 10.1016/S1471-4914(00)01909-2 11286782

[22] Hong S Beja-Glasser VF Nfonoyim BM Frouin A Li S Ramakrishnan S. Complement and microglia mediate early synapse loss in Alzheimer mouse models. Science. (2016) 352(6286):712–6. doi: 10.1126/science.aad8373 PMC5094372 27033548

[23] Pekna M Pekny M. The complement system: a powerful modulator and effector of astrocyte function in the healthy and diseased central nervous system. Cells (2021) 10(7):1812. doi: 10.3390/cells10071812 34359981 PMC8303424

[24] Werneburg S Jung J Kunjamma RB Ha SK Luciano NJ Willis CM. Targeted complement inhibition at synapses prevents microglial synaptic engulfment and synapse loss in demyelinating disease. Immunity. (2020) 52(1):167–82.e7. doi: 10.1016/j.immuni.2019.12.004 31883839 PMC6996144

[25] Miller SD Karpus WJ. Experimental autoimmune encephalomyelitis in the mouse. Curr Protoc Immunol (2007) Chapter 15:Unit 15.1. doi: 10.1002/0471142735.im1501s77 PMC2915550 18432984

[26] DiSano KD Linzey MR Royce DB Pachner AR Gilli F. Differential neuro-immune patterns in two clinically relevant murine models of multiple sclerosis. J neuroinflamma (2019) 16(1):109. doi: 10.1186/s12974-019-1501-9 PMC6532235 31118079

[27] Tsunoda I Fujinami RS. Neuropathogenesis of theiler’s murine encephalomyelitis virus infection, an animal model for multiple sclerosis. J neuroimmune pharmacol: Off J Soc NeuroImmune Pharmacol (2010) 5(3):355–69. doi: 10.1007/s11481-009-9179-x PMC2888670 19894121

[28] Miller SD Vanderlugt CL Begolka WS Pao W Yauch RL Neville KL. Persistent infection with theiler’s virus leads to CNS autoimmunity *via* epitope spreading. Nat Med (1997) 3(10):1133–6. doi: 10.1038/nm1097-1133 9334726

[29] Gilli F Royce DB Pachner AR. Measuring progressive neurological disability in a mouse model of multiple sclerosis. J Vis Exp (2016) (117):54616. doi: 10.3791/54616. PMC5226227 27911409

[30] Gilli F Li L Pachner AR. The immune response in the CNS in theiler’s virus induced demyelinating disease switches from an early adaptive response to a chronic innate-like response. J neurovirol (2016) 22(1):66–79. doi: 10.1007/s13365-015-0369-4 26260496

[31] Gilli F Chen X Pachner AR Gimi B. High-resolution diffusion tensor spinal cord MRI measures as biomarkers of disability progression in a rodent model of progressive multiple sclerosis. PloS One (2016) 11(7):e0160071. doi: 10.1371/journal.pone.0160071 27467829 PMC4965026

[32] Bjornevik K Cortese M Healy BC Kuhle J Mina MJ Leng Y. Longitudinal analysis reveals high prevalence of Epstein-Barr virus associated with multiple sclerosis. Science (2022) 375(6578):296–301. doi: 10.1126/science.abj8222 35025605

[33] Lanz TV Brewer RC Ho PP Moon JS Jude KM Fernandez D. Clonally expanded B cells in multiple sclerosis bind EBV EBNA1 and GlialCAM. Nature (2022) 603(7900):321–7. doi: 10.1038/s41586-022-04432-7. PMC9382663 35073561

[34] Li L Narayan K Pak E Pachner AR. Intrathecal antibody production in a mouse model of Lyme neuroborreliosis. J neuroimmunol (2006) 173(1-2):56–68. doi: 10.1016/j.jneuroim.2005.11.019 16387369

[35] DiSano KD Royce DB Gilli F Pachner AR. Central nervous system inflammatory aggregates in the theiler’s virus model of progressive multiple sclerosis. Front Immunol (2019) 10:1821. doi: 10.3389/fimmu.2019.01821 31428102 PMC6687912

[36] Shahi SK Freedman SN Dahl RA Karandikar NJ Mangalam AK. Scoring disease in an animal model of multiple sclerosis using a novel infrared-based automated activity-monitoring system. Sci Rep (2019) 9(1):19194. doi: 10.1038/s41598-019-55713-7 31844134 PMC6915774

[37] Pachner AR Li L Narayan K. Intrathecal antibody production in an animal model of multiple sclerosis. J neuroimmunol (2007) 185(1-2):57–63. doi: 10.1016/j.jneuroim.2007.01.017 17343922

[38] Litvinchuk A Wan YW Swartzlander DB Chen F Cole A Propson NE. Complement C3aR inactivation attenuates tau pathology and reverses an immune network deregulated in tauopathy models and alzheimer’s disease. Neuron. (2018) 100(6):1337–53.e5. doi: 10.1016/j.neuron.2018.10.031 30415998 PMC6309202

[39] Hester CG Frank MM. Complement activation by IgG containing immune complexes regulates the interaction of C1q with its ligands. Mol Immunol (2019) 116:117–30. doi: 10.1016/j.molimm.2019.10.004 31634815

[40] Jin YH Kim CX Huang J Kim BS. Infection and activation of B Cells by Theiler's Murine Encephalomyelitis Virus (TMEV) leads to autoantibody production in an infectious model of multiple sclerosis. Cells (2020) 9(8):1787. doi: 10.3390/cells9081787. PMC7465974 32727036

[41] Omura S Sato F Park AM Fujita M Khadka S Nakamura Y. Bioinformatics analysis of gut microbiota and CNS transcriptome in virus-induced acute myelitis and chronic inflammatory demyelination; potential association of distinct bacteria with CNS IgA upregulation. Front Immunol (2020) 11:1138. doi: 10.3389/fimmu.2020.01138 32733435 PMC7358278

[42] Schafer DP Lehrman EK Kautzman AG Koyama R Mardinly AR Yamasaki R. Microglia sculpt postnatal neural circuits in an activity and complement-dependent manner. Neuron. (2012) 74(4):691–705. doi: 10.1016/j.neuron.2012.03.026 22632727 PMC3528177

[43] Morgan BP Gommerman JL Ramaglia V. An “Outside-in” and “Inside-out” consideration of complement in the multiple sclerosis brain: Lessons from development and neurodegenerative diseases. Front Cell Neurosci (2020) 14:600656. doi: 10.3389/fncel.2020.600656 33488361 PMC7817777

[44] Michailidou I Naessens DM Hametner S Guldenaar W Kooi EJ Geurts JJ. Complement C3 on microglial clusters in multiple sclerosis occur in chronic but not acute disease: Implication for disease pathogenesis. Glia. (2017) 65(2):264–77. doi: 10.1002/glia.23090 PMC5215693 27778395

[45] Libbey JE Cusick MF Doty DJ Fujinami RS. Complement components are expressed by infiltrating Macrophages/Activated microglia early following viral infection. Viral Immunol (2017) 30(5):304–14. doi: 10.1089/vim.2016.0175 PMC5467122 28402228

[46] Carpanini SM Torvell M Morgan BP. Therapeutic inhibition of the complement system in diseases of the central nervous system. Front Immunol (2019) 10:362. doi: 10.3389/fimmu.2019.00362 30886620 PMC6409326

[47] Absinta M Maric D Gharagozloo M Garton T Smith MD Jin J. A lymphocyte-microglia-astrocyte axis in chronic active multiple sclerosis. Nature. (2021) 597(7878):709–14. doi: 10.1038/s41586-021-03892-7 PMC8719282 34497421

[48] Szalai AJ Hu X Adams JE Barnum SR. Complement in experimental autoimmune encephalomyelitis revisited: C3 is required for development of maximal disease. Mol Immunol (2007) 44(12):3132–6. doi: 10.1016/j.molimm.2007.02.002 PMC1986644 17353050

[49] Benoit ME Hernandez MX Dinh ML Benavente F Vasquez O Tenner AJ. C1q-induced LRP1B and GPR6 proteins expressed early in Alzheimer disease mouse models, are essential for the C1q-mediated protection against amyloid-β neurotoxicity. J Biol Chem (2013) 288(1):654–65. doi: 10.1074/jbc.M112.400168 PMC3537064 23150673

[50] Alawieh A Langley EF Weber S Adkins D Tomlinson S. Identifying the role of complement in triggering neuroinflammation after traumatic brain injury. J neurosci: Off J Soc Neurosci (2018) 38(10):2519–32. doi: 10.1523/JNEUROSCI.2197-17.2018 PMC5858594 29437855

[51] Mevorach D Mascarenhas JO Gershov D Elkon KB. Complement-dependent clearance of apoptotic cells by human macrophages. J Exp Med (1998) 188(12):2313–20. doi: 10.1084/jem.188.12.2313 PMC2212421 9858517

[52] Taylor PR Carugati A Fadok VA Cook HT Andrews M Carroll MC. A hierarchical role for classical pathway complement proteins in the clearance of apoptotic cells in vivo. J Exp Med (2000) 192(3):359–66. doi: 10.1084/jem.192.3.359 PMC2193213 10934224

[53] Liddelow SA Guttenplan KA Clarke LE Bennett FC Bohlen CJ Schirmer L. Neurotoxic reactive astrocytes are induced by activated microglia. Nature. (2017) 541(7638):481–7. doi: 10.1038/nature21029 PMC5404890 28099414

[54] Färber K Cheung G Mitchell D Wallis R Weihe E Schwaeble W. C1q, the recognition subcomponent of the classical pathway of complement, drives microglial activation. J Neurosci Res (2009) 87(3):644–52. doi: 10.1002/jnr.21875 PMC2635544 18831010

[55] Nataf S Carroll SL Wetsel RA Szalai AJ Barnum SR. Attenuation of experimental autoimmune demyelination in complement-deficient mice. J Immunol (2000) 165(10):5867–73. doi: 10.4049/jimmunol.165.10.5867 11067947

[56] Boos LA Szalai AJ Barnum SR. Murine complement C4 is not required for experimental autoimmune encephalomyelitis. Glia. (2005) 49(1):158–60. doi: 10.1002/glia.20093 15390104

