# Supplementary figures and images for: Divergent complement system activation in two clinically distinct murine models of multiple sclerosis
Source: Front Immunol. 2022 Jul 26;13:924734. doi: 10.3389/fimmu.2022.924734 (PMC9360327; doi:10.3389/fimmu.2022.924734)

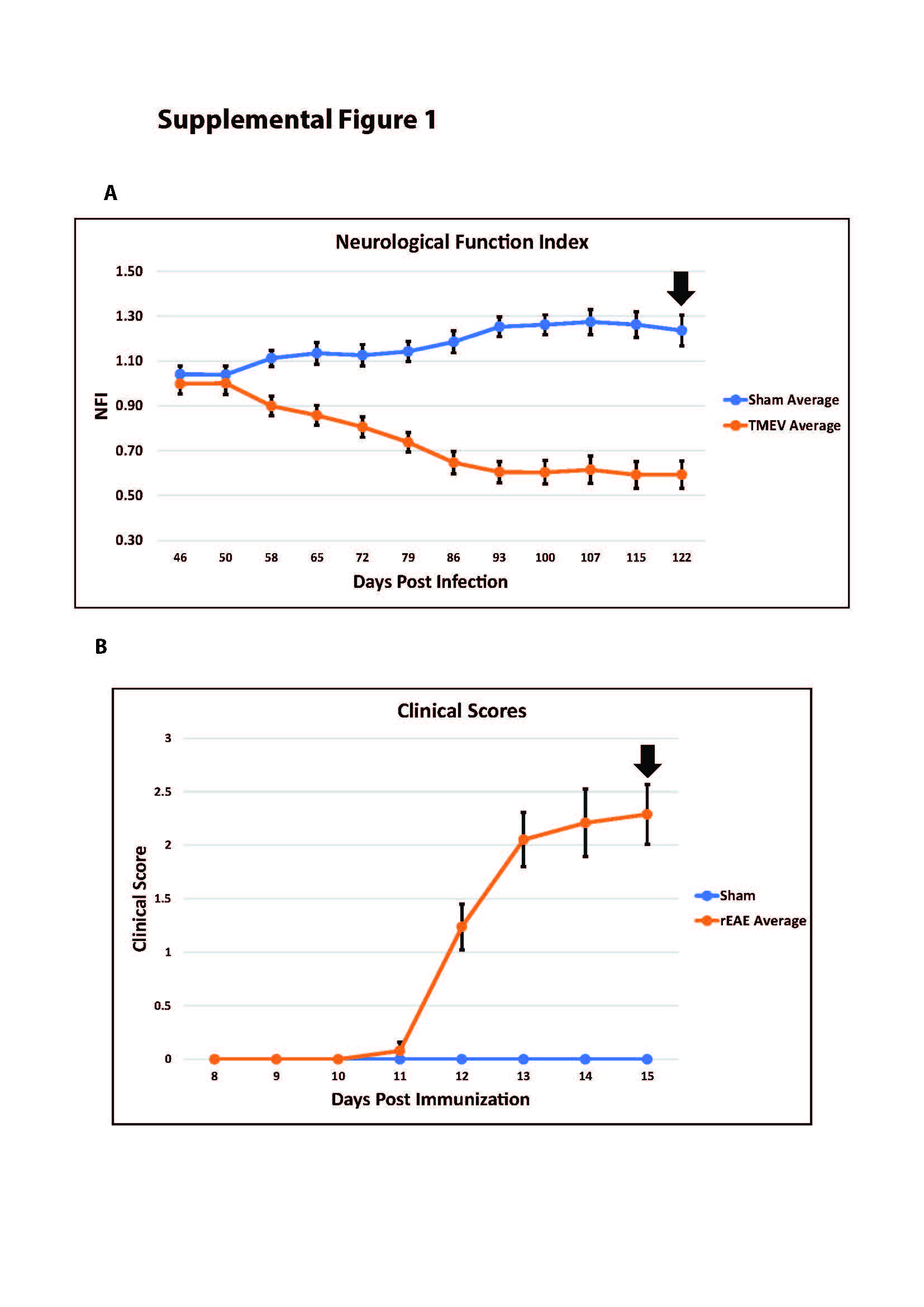

Supplement: Supplementary Figure 1 — Clinical disease progression in TMEV-IDD and rEAE mice included in this study. Disease progression of TMEV-IDD (A) and rEAE (B) over their respective monitor periods post-induction. Each plot indicates the time points chosen for analysis of CNS tissue: TMEV-IDD mice were necropsied at around 120 dpi to capture the chronic progressive phase of the disease. rEAE mice were necropsied around 15 dpimm to capture the acute phase of the first relapse. (A) The Rotarod Test was performed on 26 TMEV-infected mice and 10 age-matched sham controls over an average of 120 dpi. Eight were excluded from the experiment because they tested TMEV-negative at the end of their follow-up. Rotarod data were expressed as a neurological function index (NFI) (29). (B) Clinical symptoms score (CSS) from day 1 to day 15 dpimm of 20 rEAE mice. Four mice were withdrawn from the experiment because not symptomatic at the end of the follow up. The circles and confidence bars represent the mean and the SEM of the (A) NFI or (B) CSS of the entire group of mice for each day p.i. Blue circles represent diseased mice and orange squares represent sham-treated controls. [file Image_1.jpeg]

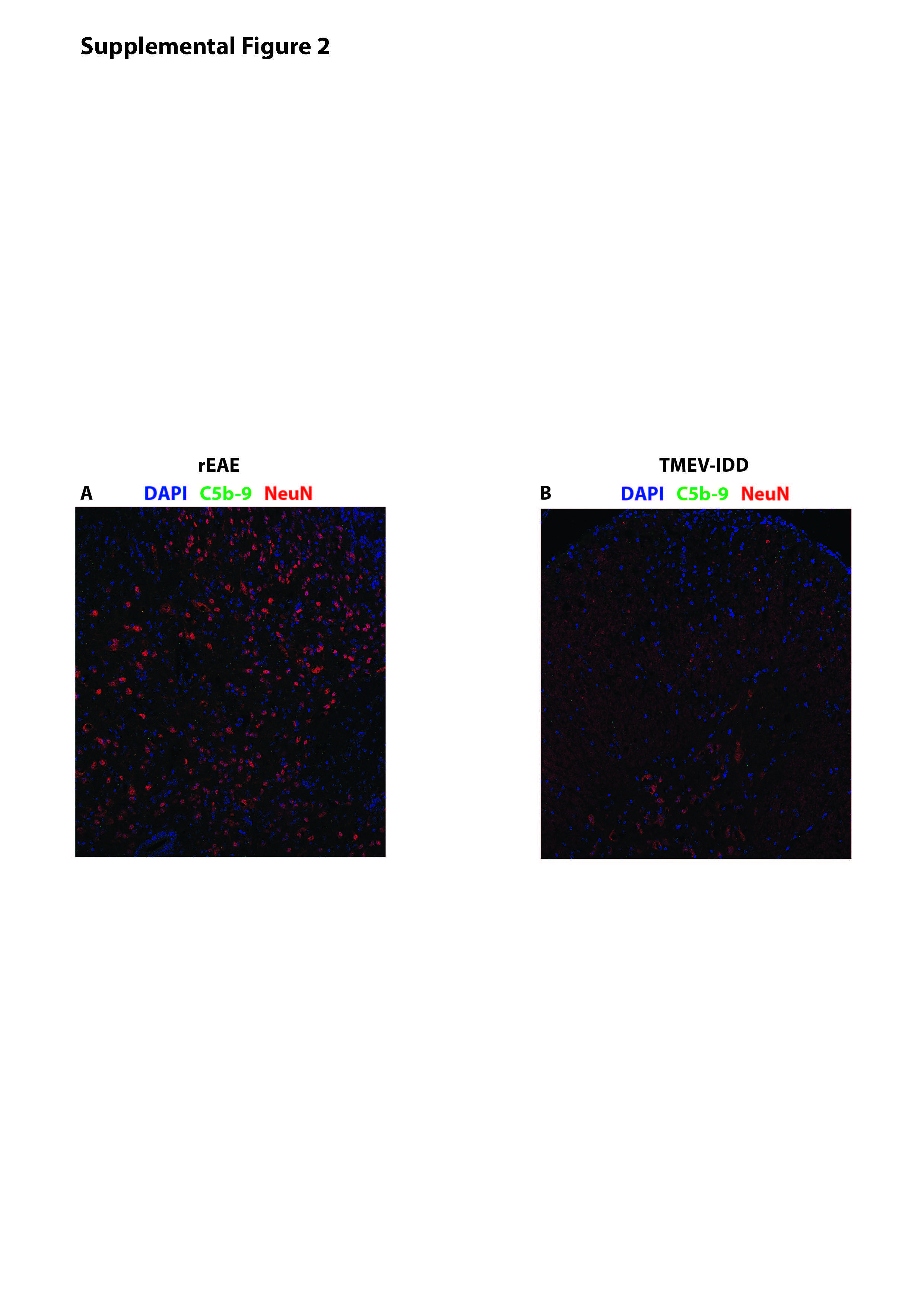

Supplement: Supplementary Figure 2 — Colocalization of C5b-9 and NeuN in the spinal cord of TMEV-IDD and rEAE mice. TMEV-IDD and rEAE mice were necropsied at ~120 dpi (chronic stage) and ~15 dpimm (acute relapse), respectively. Spinal cords were harvested for immunohistochemical analyses of the late complement component C5b-9 and the neuronal marker NeuN. In both (A) rEAE and (B) TMEV-IDD mice, we found that C5b-9 (green) does not colocalize with NeuN (red), suggesting that, in these mice, there is no triggering of the cytotoxic component of the complement system. Images are representative of 3 to 4 mice per group. Representative z stacks are shown and the scale bar=100µM. [file Image_2.jpeg]
